# Chronic polycyclic aromatic hydrocarbon exposure causes DNA damage and genomic instability in lung epithelial cells

**DOI:** 10.18632/oncotarget.20891

**Published:** 2017-09-15

**Authors:** Hongzhen Bai, Min Wu, Hongjian Zhang, Guping Tang

**Affiliations:** ^1^ State Key Laboratory of Industrial Control Technology, College of Control Science and Engineering, Zhejiang University, Hangzhou 310028, China; ^2^ Institute of Chemical Biology and Pharmaceutical Chemistry, Zhejiang University, Hangzhou 310028, China

**Keywords:** human lung epithelial cells, polycyclic aromatic hydrocarbon, genotoxicity, cell cycle, apoptosis

## Abstract

Cell exposure to atmospheric polycyclic aromatic hydrocarbons (PAHs) is closely associated with DNA damage and genomic instability. We assessed the mechanisms of chronic and acute PAH exposure-induced genotoxicity in two human lung epithelial cell lines, A549 and NC-H1975. We sampled atmospheric PAHs at the Xixi Campus, Zhejiang University in Hangzhou, China during August (non-haze episode) and November (haze episode) 2015. We identified vehicle emissions as a dominant anthropogenic PAH source in our study. PAHs were extracted according to the United States Environmental Protection Agency Method TO-13A. We found that chronic PAH exposure saturated lung cell xenobiotic metabolic pathways, promoting intercellular reactive oxygen species production and accumulation. Chronic alteration of the cellular redox status resulted in DNA damage and genomic instability. Chronic PAH exposure also perturbed the cellular DNA damage response, inducing S phase arrest and inhibiting apoptosis. Dysregulation of PAH metabolism and the DNA damage response altered cellular homeostasis and increased cell susceptibility to subsequent PAH exposures, thereby enhancing the likelihood of genomic mutation and instability.

## INTRODUCTION

Atmospheric particulate matter (PM) pollution is a growing concern worldwide due to its negative effects on air quality, climate change, and human health [1–6]. Atmospheric polycyclic aromatic hydrocarbons (PAHs) are considered priority environmental pollutants due to their carcinogenicity and mutagenicity [7–9]. Procarcinogens, including PAHs, do not directly induce genotoxicity [10]. Instead, PAHs must be metabolically transformed into more reactive metabolites by a series of xenobiotic-metabolizing enzymes (XMEs), including cytochrome P450 (CYP) 1A1/1B1, epoxide hydrolase (EH), and aldo-keto reductase (AKR). PAH activation involves the formation of phenols, catechols, quinones, diol-epoxides, o-quinones, and radical cations [11–13], which can react with DNA to form DNA adducts, leading to deletions, fusions, translocations, or aneuploidy. Mutation risk depends not only on the DNA damage level, but also the effectiveness of the DNA damage response (DDR) [14, 15]. The DDR is a complex signal transduction network that activates processes coordinating cell cycle progression, DNA repair, DNA replication, and apoptosis. The carcinogenic PAHs can induce DNA damage in various human cell lines [16, 17], activating DDR pathways regulated by P53 and its downstream gene products to regulate and maintain genomic stability [18–20].

PAHs bound to PM <2.5μm in diameter (PM2.5) are actually a complex mixture of multiple PAH compounds. While studies have explored the mutagenic and potentially carcinogenic activities of PM2.5-bound PAHs [21–24], little is known about the chronic genotoxic and physiological results of long-term PAH exposure. In this study, we first characterized long-term PAH exposure by collecting air samples during non-haze and haze episodes, and then simulated long-term PAH exposure in the human lung epithelial cell lines, A549 and NCI-H1975. We found that chronic low-dose PAH exposure dysregulated PAH metabolism and the DDR, enhancing DNA mutation susceptibility. With further exposure to toxic environmental stimuli, this sensitivity could be an important contributor to pathological transformation.

## RESULTS

### Atmospheric PAH concentrations increase during seasonal haze episodes

**Figure 1:**
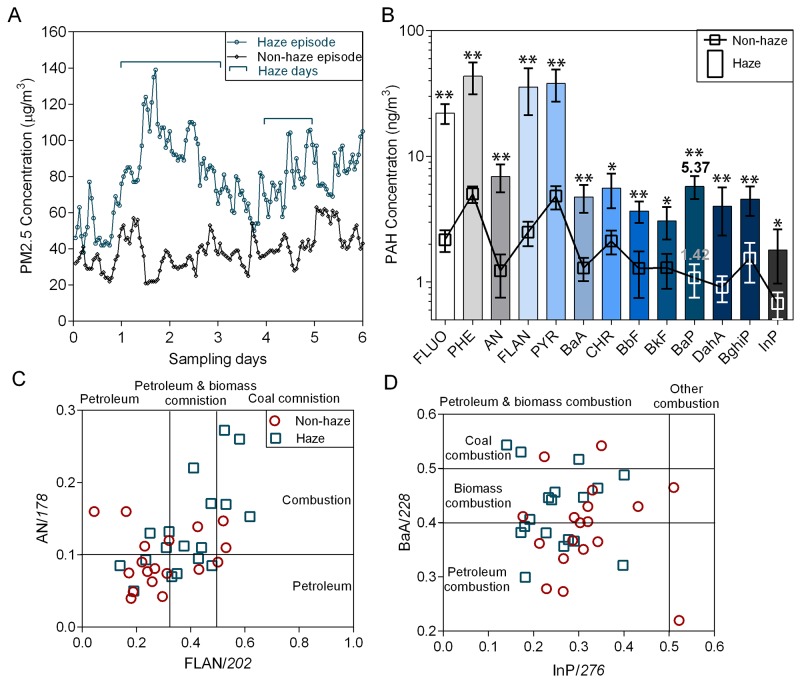
Atmospheric PAHs during haze and non-haze episodes Average PM2.5 **(A)** and PM2.5-bound PAH **(B)** concentrations during both episodes (n=5). *p<0.05, **p<0.01 via Student’s t-test. PAH sources were determined using diagnostic ratios: An/178 (Y axis) and Flan/202 (X axis) **(C)**; BaA/228 (Y axis) and InP/276 (X axis) **(D)** Fluo: fluorene; Phe: phenanthrene; An: anthracene; Fla: fluoranthene; Pyr: pyrene; BaA: benzo [a] anthracene; Chr: chrysene; BbF: benzo [b] fluoranthene; BkF: benzo [k] fluoranthene; BaP: benzo [a] pyrene; InP: indeno [1,2,3-cd] pyrene; DaA: dibenz [a,h] anthracene; BghiP: benzo [ghi] perylene.

Concentrations of PM2.5 and PM2.5-bound PAHs during the sampling periods are shown in Figure 1A–1B. Daily PM2.5 concentration averages were 33–52 μg/m^3^ during the non-haze episode and 65–124 μg/m^3^ during the haze episode. Similarly, concentrations of individual PAHs increased with haze (Figure 1B). The average concentration of total PAHs increased from 42–87 ng/m^3^ during the non-haze episode to 168–307 ng/m^3^ during the haze (p<0.01). The PM2.5-bound PAHs increase indicated that one or more temporary sources acted during the haze episode. To evaluate emission sources, proportions of individual PAHs and their isomers were used as diagnostic ratios. The diagnostic ratios of anthracene (An)/178, fluoranthene (Flan)/202, benzo [a] anthracene (BaA)/228 and indeno [1,2,3-cd] pyrene (InP)/276 were used to characterize PAH sources [25]. The scattered distribution of diagnostic ratios (Figure 1C–D) indicated that traffic emission (vehicle exhaust) was a dominant anthropogenic PAH source. Biomass combustion was another PAH source during the haze episode. Outdated agricultural waste treatment methods, including straw burning in China, have contributed noticeably to the occurrence of seasonal haze and increased atmospheric PAHs, especially in some agricultural provinces.

Although toxicological studies have demonstrated that atmospheric PAHs induce acute cytotoxicity and mutagenicity in many cell lines [26–28], the biological effects of long-term, low-dose PAH exposure, followed by high-dose exposure, are not well understood. In our study, each cell line was divided into two groups. Group 1 was defined as the 0 generation (0 G) and was directly treated with PAHs extracted from haze. Group 2 was defined as the 10 generation (10 G) and underwent chronic exposure before being treated with the PAH extract. The 10 G was first incubated in low-dose PAH-contaminated culture medium for 10 generations, and was then exposed to the haze PAH extract. PAH-induced genotoxic effects were assessed in both groups.

### Chronic PAH exposure increases cell mortality

**Figure 2:**
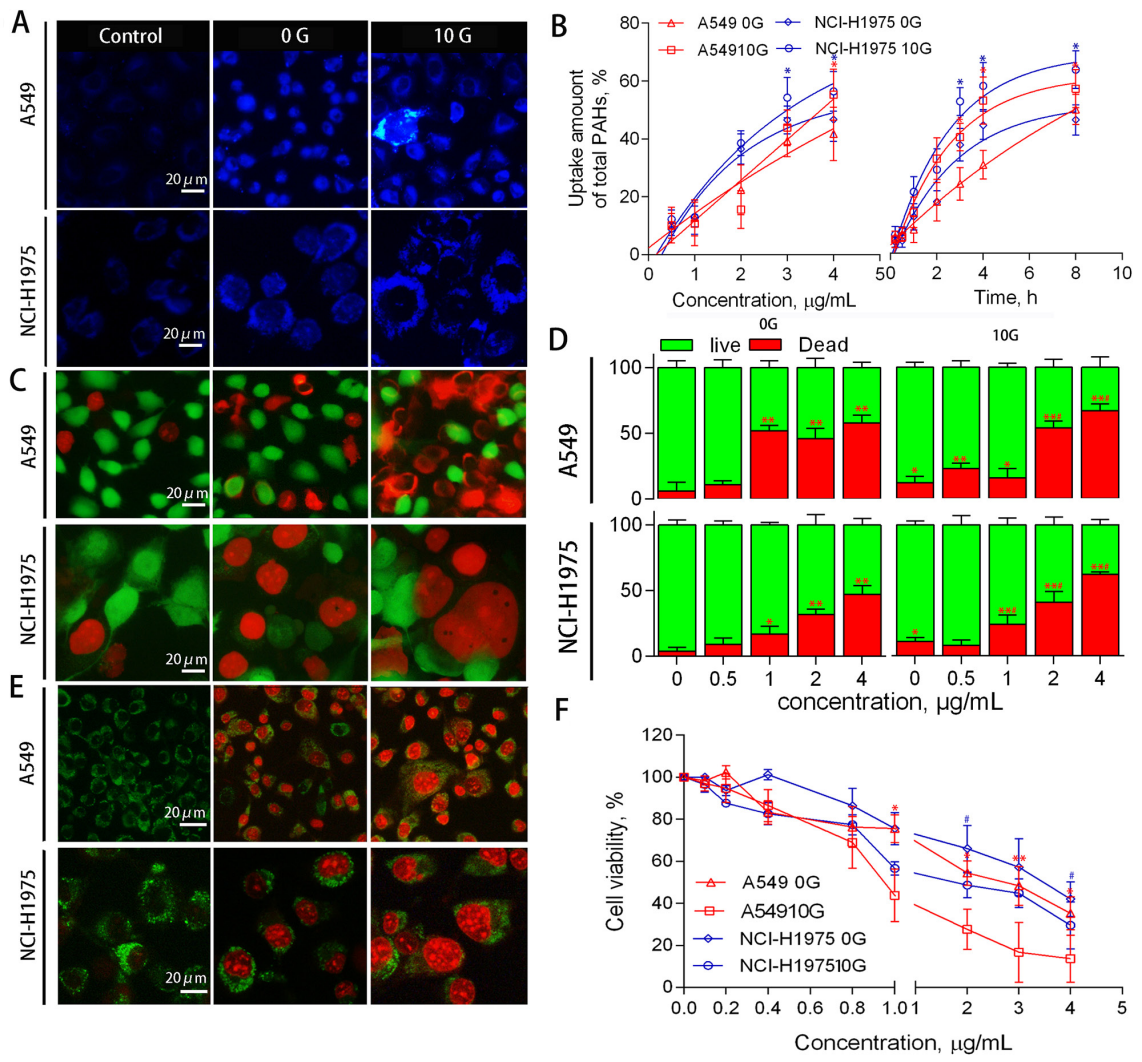
A549 and NCI-H1975 cell PAH uptake, viability, and death phenotype Representative fluorescent microscopy images of PAH uptake **(A)** Response curves showing PAH uptake based on PAH concentration (left) and exposure duration (right) (n=3). *p<0.05 compared to 0 G cells **(B)** Representative fluorescent microscopy images of live/dead cells detected using calcein-AM and PI **(C)** Histograms of live/dead cells as determined by flow cytometry (n=3) **(D)** *p<0.05, **p<0.01 compared to controls; #p<0.05 compared to untreated 10 G cells. Representative fluorescent microscopy images showing cell death phenotypes using an apoptosis/necrosis staining kit **(E)** Dead 0 G cells exhibited classic apoptosis morphological features, including pyknosis, chromatin condensation, and karyorrhexis. Some dead 10 G cells presented necrotic characteristics, such as oncosis and cell membrane rupture. Cells were treated with PAH extract for 24 h, and cell viability was detected via MTT assay (n=3) **(F)** *p<0.05 compared to controls (0.5% DMSO-treated cells).

After chronic PAH exposure for 30 d, both A549 and NCI-H1975 10 G cells appeared normal in morphology. 10 G and 0 G cells were simultaneously exposed to particulate PAHs extracted from haze. Fluorescence-based observations of cellular uptake (Figure 2A) indicated that morphology differed between the two groups following treatment. 0 G cells were small in size with regular nuclei and granular cytoplasms, while 10 G cells divided and cracked with atypical nuclei and cytoplasm. Uptake-time/dose-response curves showed that PAH accumulation was 16–20% (p<0.05) higher in 10 G than in 0 G cells (Figure 2B). After cellular uptake for 24 h, live vs. dead cells were detected using Calcein-AM and PI. Fluorescence-based observations (Figure 2C) and flow cytometric analyses (Figure 2D) showed that A549 and NCI-H1975 10 G cell mortalities increased by 22–34% and 11–18% (p<0.05), respectively. MTT assay showed that PAH half-maximum inhibiting concentrations (IC50 values) were 1.6–2.1-fold higher (p<0.05) in 0 G compared to 10 G cells (Figure 1F). Cells were stained to detect apoptosis/necrosis using Hoechst solution and PI (Figure 1E). Dead 0 G cells exhibited the morphologic features of apoptosis, while some dead 10 G cells presented necrotic characteristics, such as oncosis and cell membrane rupture [29]. Together, these results suggest that chronic PAH exposure had latent effects on cell viability and physiology, potentiating cell mortality and inducing necrosis with further PAH treatment. Because PAH-induced cytotoxicity was associated with reactive oxygen species (ROS) metabolites, latent effects of chronic PAH exposure might be associated with xenobiotic metabolizing pathways and PAH-ROS biotransformation.

### Chronic PAH exposure saturates xenobiotic metabolic pathways

**Figure 3:**
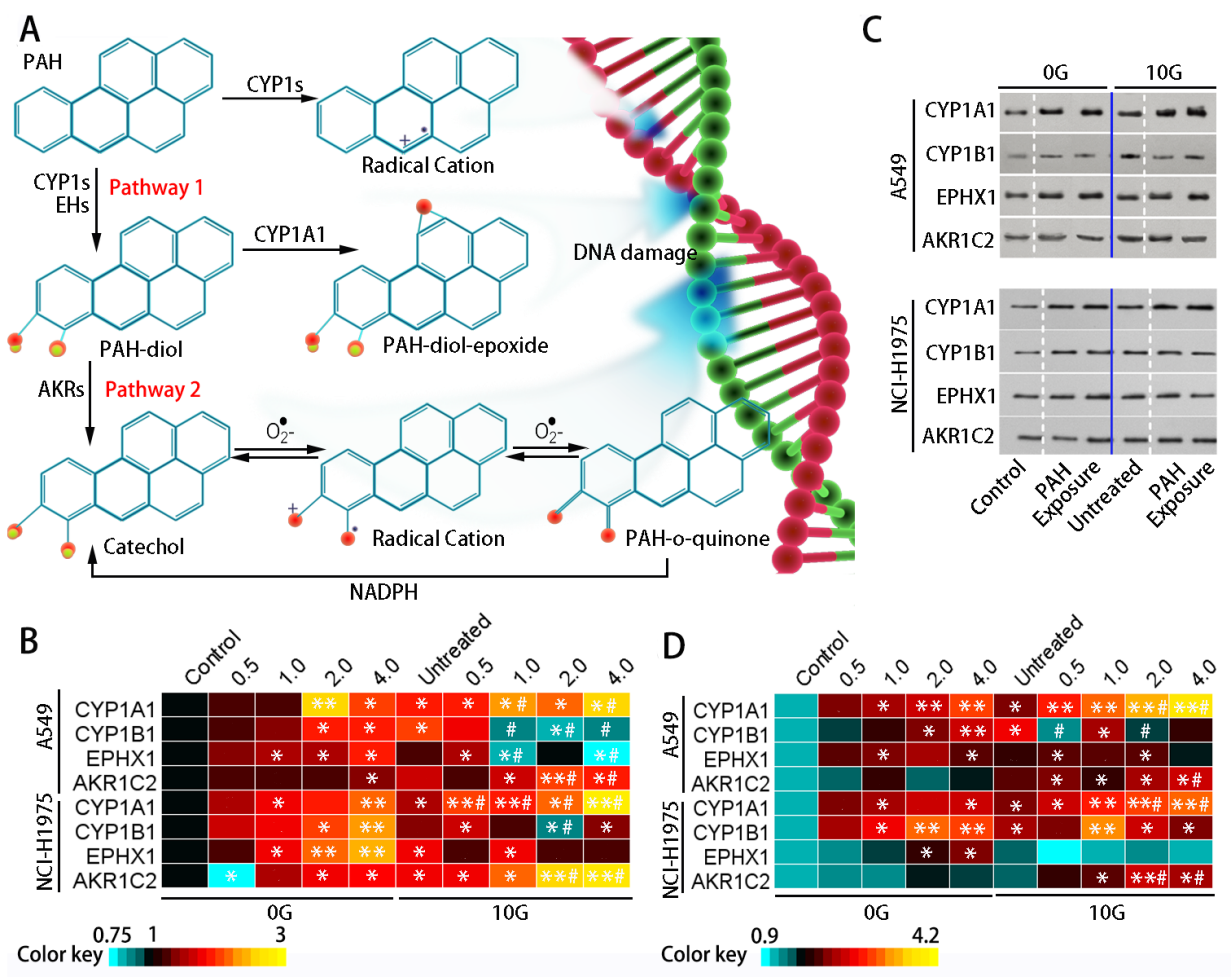
PAH metabolic pathways and XME levels Metabolic activation of PAHs **(A)** Pathway 1: CYP1s activate PAHs to form PAH-epoxides on the bay region benzo-ring; subsequent EPHX1-mediated hydrolysis results in PAH-dihydrodiol (PAH-diol) formation. The dihydrodiols can undergo a secondary epoxidation to form bay region PAH-diol-epoxides. Pathway 2: Members of the AKR superfamily can transform PAH-diol to the corresponding PAH-o-quinone. Heatmap showing relative (fold change over controls) CYP1A1, CYP1B1, EPHX1, and AKR1C2 expression in the two groups as determined via qRT-PCR (n=3) **(B)** Representative western blotting results showing CYP1A1, CYP1B1, EPHX1, and AKR1C2 levels **(C)** Heatmap showing relative (fold change over controls) CYP1A1, CYP1B1, EPHX1, and AKR1C2 protein levels (n=3) **(D)** *p<0.05, **p<0.01 compared to controls; #p<0.05 compared to untreated 10 G cells.

Parental PAH molecules are considered pro-cytotoxic substances. Biotransformation of a PAH into a PAH-ROS metabolite contributes to the detrimental effect. PAH-ROS biotransformation involves two major pathways: CYP1s-EPHX1 and CYP1s-AKR (Figure 3A). CYP1s-EPHX1 catalyzes the transformation of PAHs into PAH-diol-epoxides, the ultimate carcinogenic metabolites. The AKR superfamily shunts PAH-diols to the corresponding o-quinone, a highly reactive ROS, and the AKR1C2 isoform preferentially oxidizes PAH-diols in human cells [30].

**Figure 4:**
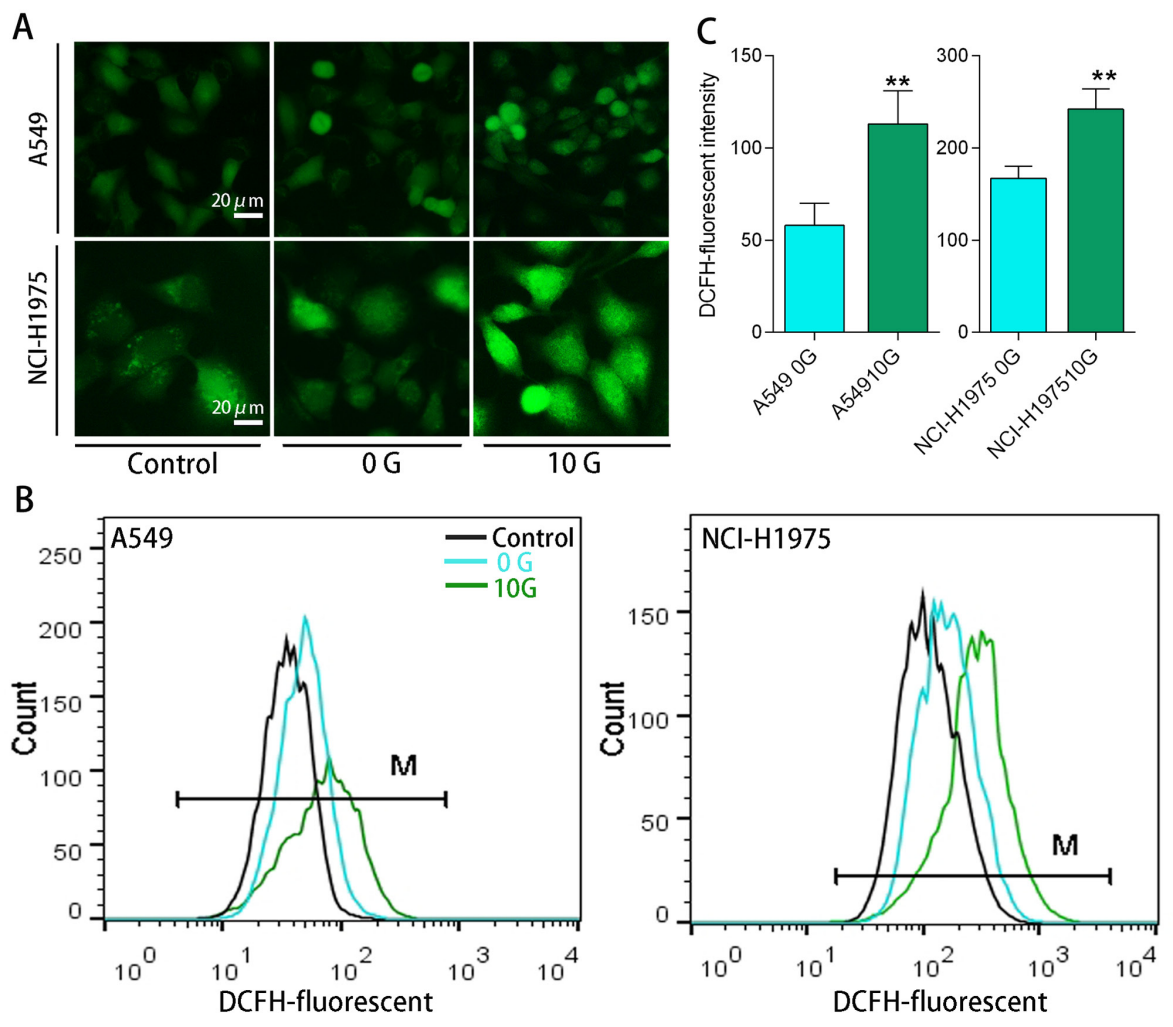
Intracellular ROS levels determined using an H2DCFDA ROS probe Representative fluorescent microscopy images showing intracellular ROS in A549 and NCI-H1975 cells **(A)** Representative DCFH fluorescence intensities as determined using flow cytometry **(B)** DCFH fluorescent intensity analysis results (n=3) **(C)** **p<0.01 compared with 0 G cells.

To investigate PAH metabolism in the two treatment groups, we monitored expression of the primary XMEs, including CYP1A1, CYP1B1, EPHX1, and AKR1C2. qRT-PCR results (Figure 3B) showed that CYP1A1, CYP1B1, and EPHX1 exhibited dose-dependent upregulation (p<0.05) in the 0 G group after 24 h PAH exposure. CYP1A1 and EPHX1 activation was increased compared to CYP1B1. Treatment did not change AKR1C2 levels in the 0 G group, possibly because low molecular weight PAHs were abundant in the extract, and were readily converted by CYP1s-EPHX1. With chronic PAH exposure, levels of these XMEs increased to different extents. CYP1s and AKR1C2 increased by approximately 1.4–2.5-fold (p<0.05) over controls, while the change of EPHX1 expression was not statistically significant. With subsequent exposure, CYP1A1 and AKR1C2 levels were increased by 2.3–3.0-fold (p<0.05) over controls. However, CYP1B1 expression decreased with increasing PAH concentration. EPHX1 expression in both cell lines was inhibited at 2.0–4.0 μg/mL PAHs (Figure 3C–3D). To evaluate intracellular ROS levels in the two treatment groups, cells were loaded with a ROS probe (DCFH-DA) (Figure 4A) and DCFH fluorescent intensity was measured using flow cytometry (Figure 4B). DCFH fluorescence intensity was higher (p<0.01) in the 10 G than the 0 G group, implying that PAHs were more readily converted to ROS in pre-treated cells (Figure 4C).

Our results suggest that XMEs are activated by acute PAH exposure, which enables cells to metabolize PAHs. However, when chronically exposed cells are subjected to high PAH exposure, their metabolic functions are dysregulated: PAH hydrolysis via CYP1s-EPHX1 is partially inhibited, while the CYP1s-AKR pathway is promoted, which may increase PAH-o-quinone formation. PAH-o-quinones can form stable DNA adducts and can undergo non-enzymatic or enzymatic redox cycles (Figure 3A) in which ROS generation is amplified multiple times.

### Chronic PAH exposure reduces genomic stability

**Figure 5:**
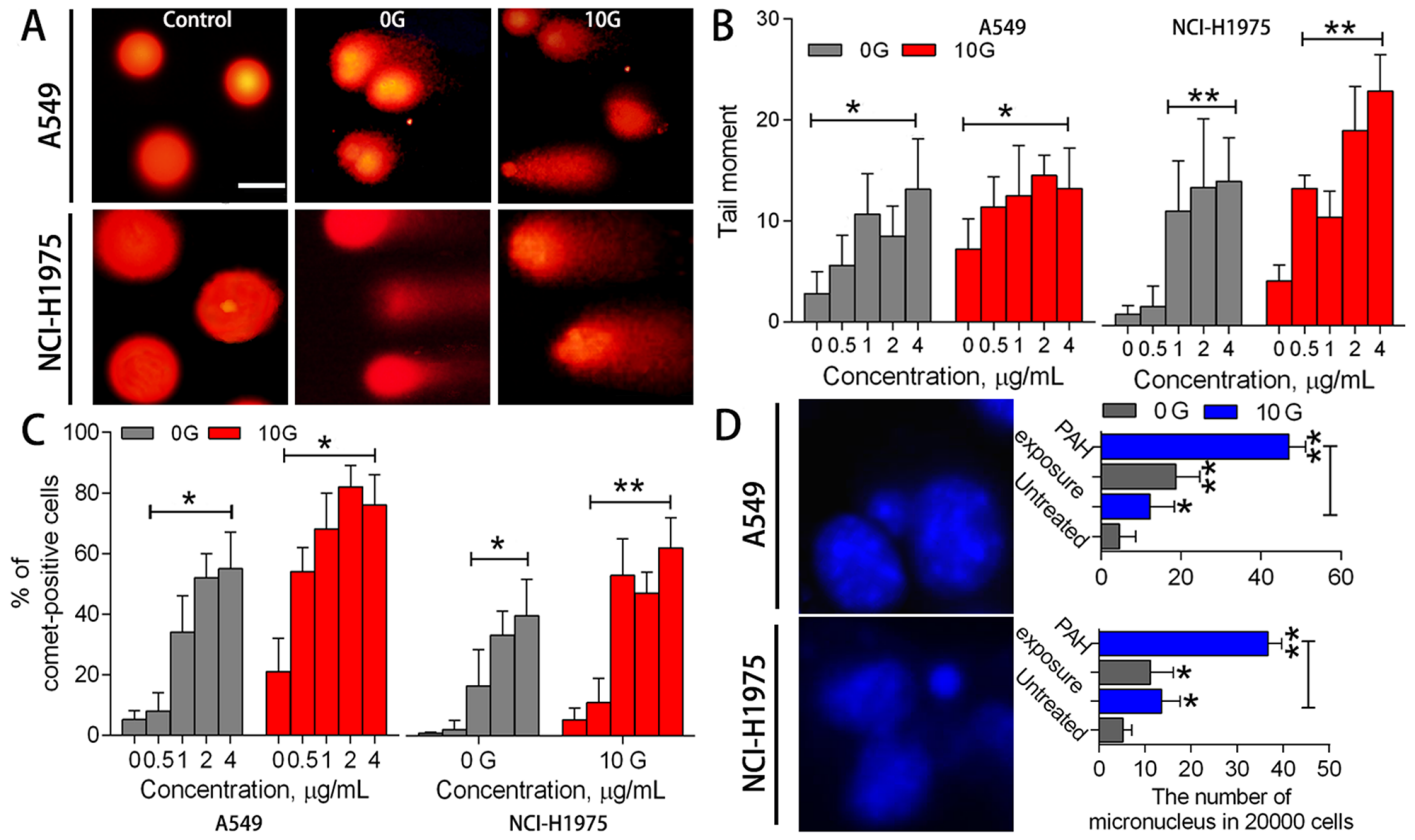
DNA damage and genomic instability assessments Representative DNA comet images in A549 and NCI-H1975 cells after PAH exposure **(A)** Controls were almost tail-negative. When cells containing damaged DNA were subjected to electrophoresis, fragmented DNA migrated towards the positive electrode, away from the nucleus. Statistical tail moment values for treated cells (n=3) **(B)** Percentages of tail-positive cells (n=3) **(C)** Representative micronucleus images (left), and numbers of micronuclei in treated and untreated samples (right; 2x10^3^ cells/sample) (n=3) **(D)** *p<0.05, **p<0.01 compared to controls.

PAH-ROS can covalently bind nucleic acids in DNA to form DNA adducts, leading to genotoxicity and inheritable DNA instability. To assess DNA lesions and genomic instability in PAH-treated cells, Comet and Micronucleus assays were performed in the two treatment groups. Comet heads in controls were intact nucleoids with concentrated DNA, and comet cell frequency was less than 5% (Figure 5A). With acute PAH treatment, tail moments were amplified (p<0.05) in a dose-dependent manner (Figure 5B), and the proportion of comet cells increased (p<0.05) at high PAH doses (Figure 5C). Figure 5D shows representative micronuclei after conventional nuclear staining, and the numbers of micronuclei formed were scored in the two treatment groups. Acute PAH treatment increased micronucleus frequency in 0G cells by 2.2–3.0-fold, with no clear dose-response relationships. Although acute PAH exposure elicited dose-dependent DNA damage, the resulting genomic instability was not always transmitted to daughter cells. In contrast, micronuclei increased in 10 G cells by 1.7–4.6-fold (p<0.05) following acute PAH exposure, even though the proportion of comet cells was negligible. This implied that chronic PAH exposure induced heritable DNA damage to some small extent, and long-term accumulation of DNA damage promoted micronucleus formation.

10 G cells exposed to the PAH extract showed reduced DNA integrity and stability. The proportion of comets in 10 G cells was 1.2–4.2-fold (p<0.05) higher than in the 0 G group, and DNA migration was more substantial. Additionally, micronuclei were increased by 10-fold (p<0.01) in 10 G cells compared to controls. In general, cells appeared more susceptible to exogenous DNA-damaging agents after chronic exposure. PAH extract-induced genome instability in pre-treated cells lead to serious DNA lesions and corresponding mutations.

### Chronic PAH exposure perturbs the DDR

**Figure 6:**
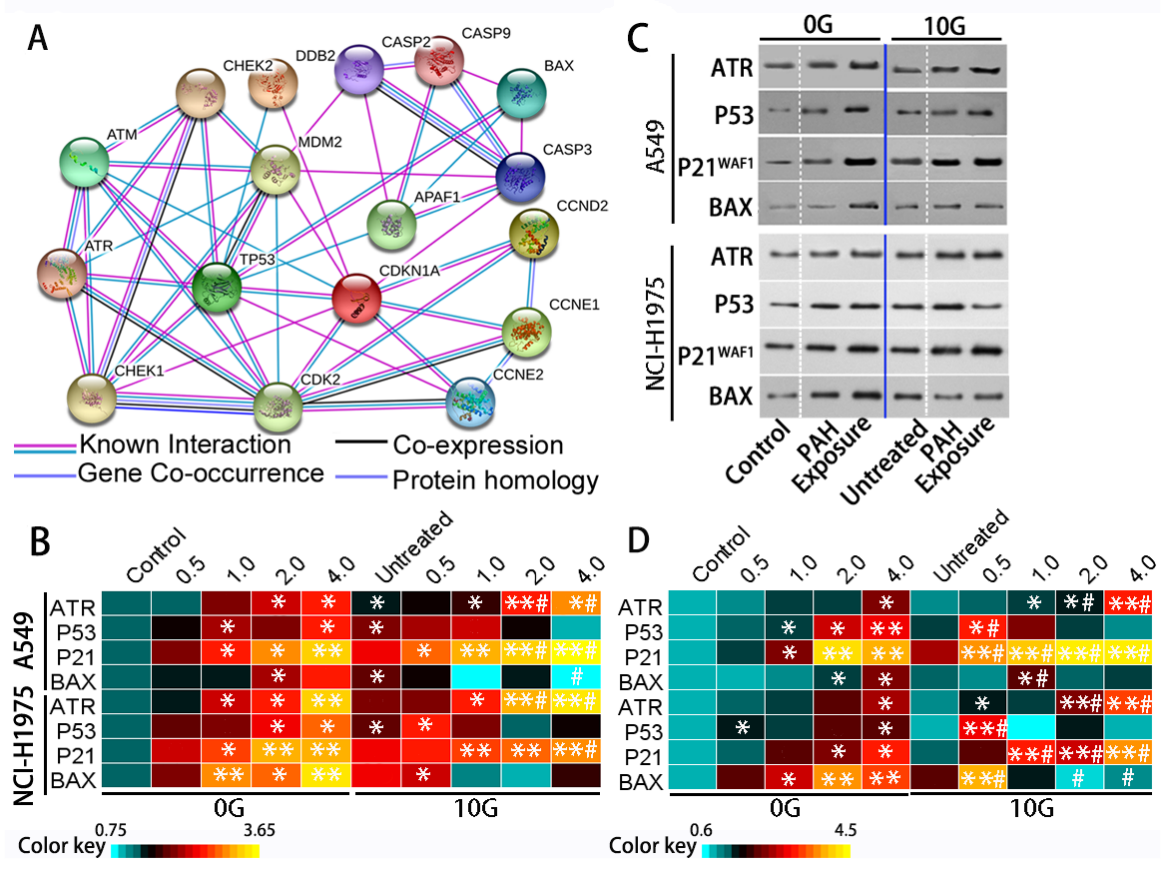
The DDR is triggered by acute and chronic PAH exposure The DDR signaling network **(A)** DNA damage checkpoints employ damage sensor proteins, such as ATM and ATR, to detect DNA damage and initiate signal transduction cascades that employ Chek1 and Chek2. Signal transducers activate P53 and inactivate cyclin-dependent kinases to inhibit cyclin-associated cell cycle progression. Apoptosis (associated with caspase and Bax) eliminates damaged or deregulated cells. Heatmap of relative (fold change over controls) ATR, P53, P21^WAF1^, and Bax expression as determined via qRT-PCR (n=3) **(B)** Representative western blotting results showing ATR, P53, P21^WAF1^, and Bax levels **(C)** Heatmap of relative (fold change over controls) ATR, P53, P21^WAF1^, and Bax protein levels (n=3) **(D)** *p<0.05, **p<0.01 compared to controls; #p<0.05 compared to untreated 10 G cells.

In response to genotoxic stresses, the DDR is indispensable for maintaining genomic integrity [31]. Interference with the DDR leads to DNA repair failure and increased genomic instability. Figure 6A shows a simplified schematic representation of the DDR networks built using STRING v10. Major DDR regulators include the phosphoinositide 3-kinase (PI3K)-related protein kinases, such as ataxia-telangiectasia mutated (ATM) and ATM and RAD3-related (ATR). ATM functions in response to rare occurrences of double-strand breaks (DSBs), while ATR is activated in response to many types of DNA damage [14]. P53 and its downstream proteins regulate key processes in cell defense following DNA damaging agent exposure, including DNA repair, cell cycle progression, and apoptosis [32].

**Figure 7:**
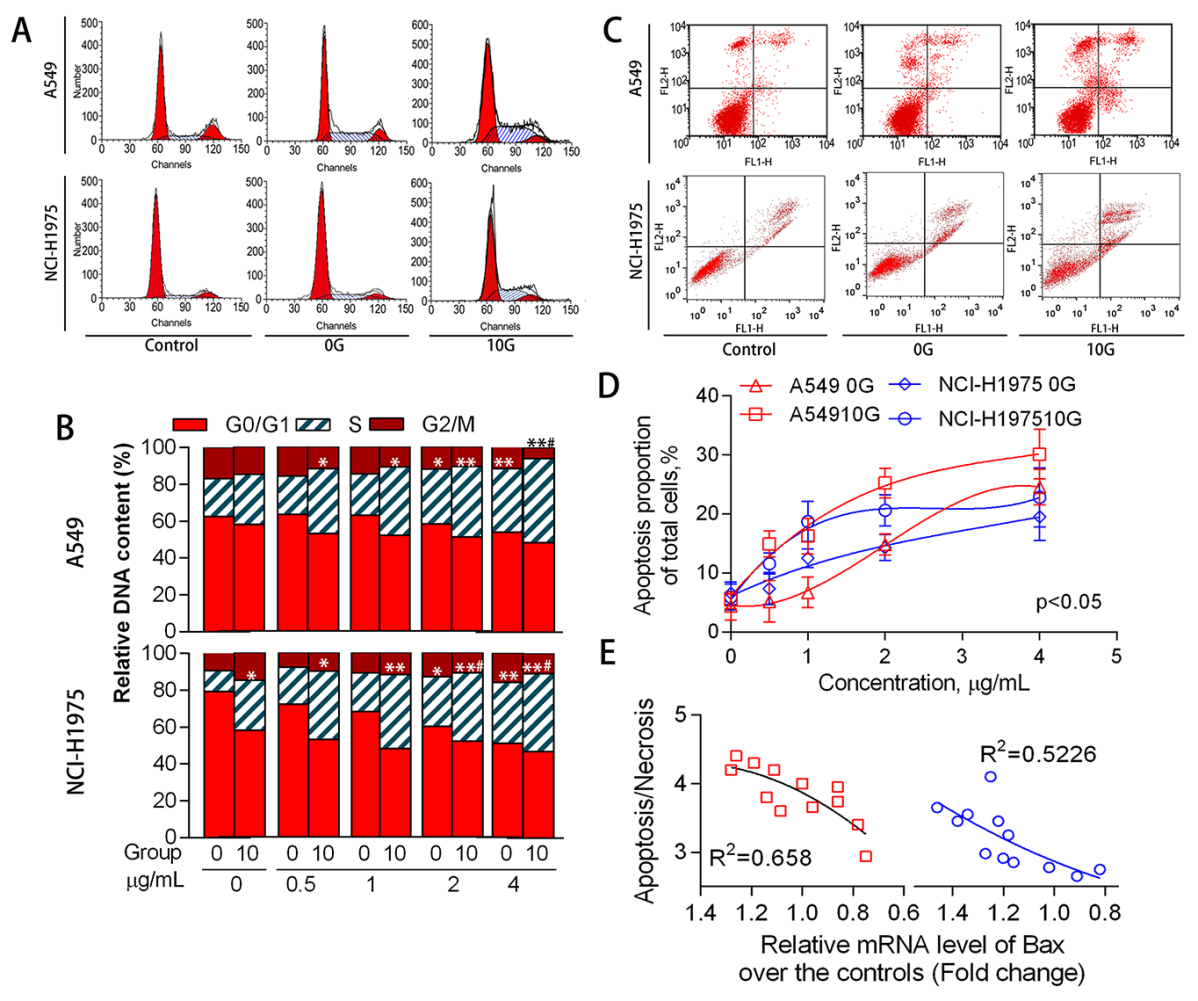
Cell cycle distribution and apoptosis as mediated by acute and chronic PAH exposure Representative cell cycle distributions **(A)** and histograms **(B)** for the different treatment groups (n=3). Representative cell apoptosis phenotypes as determined via flow cytometry **(C)** Proportions of apoptotic cells at different PAH concentrations (n=3) **(D)** Relationship between Bax expression and apoptosis/necrosis in 10 G cells **(E)** *p<0.05, **p<0.01 compared with the controls.

ATR and P53 levels increased (p<0.05) in A549 and NCI-H1975 cells with increasing PAH concentration (Figure 6B–6D). The P53 target gene, cyclin dependent kinase inhibitor 1A (P21^WAF1^), was also increased (p<0.05) compared to controls. Although P21^WAF1^ inhibits activities of cyclin/cdk family members, [33] the number of cells in S phase increased dose-dependently (p<0.05), while the number of cells in G0/G1 and G2/M phases decreased (Figure 7A–7B). In addition to cell cycle arrest, cells with irreparable DNA damage underwent apoptosis. Flow cytometric analysis (Figure 7C–D) showed that the proportion of apoptotic cells increased in a dose-dependent manner. Activation of the apoptosis-associated gene, BCL2 associated X (Bax), also increased with apoptosis.

Chronic PAH exposure detectably increased ATR levels, and P53 expression significantly increased by 0.6–2.0-fold (average 1.2-fold in parallel samples) over controls. The proportion of cells in S phase further increased to 27.4% (p<0.05), P21^WAF1^ was upregulated, and apoptosis increased by approximately 1.3-fold compared to controls. Bax expression increased by 0.6–1.6-fold over controls. These expression changes might have counteracted chronic oxidative stress and addressed genetic instability in PAH-exposed cells, but instead appeared to perturb DDR network homeostasis.

In 10 G cells, ATR was upregulated by acute exposure to PAH extracts for 24 h (p<0.05). However, P53 expression was reduced at high PAH concentrations in A549 cells and was unstable in NCI-H1975 cells. P21^WAF1^ levels increased (p<0.01) over controls, leading to S phase arrest. Bax inhibition was observed as PAH concentration rose in both A549 and NCI-H1975 cells. At the same time, the number of apoptotic cells decreased and more cells were necrotic (Figure 7E). Because cell apoptosis essentially eliminates damaged DNA, apoptosis inhibition promoted inherited genomic instability and micronucleus formation.

## DISCUSSION

Continuous hazy weather in autumn and winter has become a serious environmental issue, in part result in a surge in atmospheric PAHs. We assessed PAH-associated genotoxic effects in two human lung epithelial cell lines, A549 and NCI-H1975. Chronic PAH exposure perturbed cellular homeostasis, leading to enhanced DNA damage and genomic instability.

The metabolic activation of PAHs via XMEs leads to carcinogenicity [34]. Acute PAH exposure activated CYP1s and EPHX1, transforming the PAHs into their electrophilic diol-epoxides. PAH-diol-epoxides react with DNA to produce adducts that have been identified in lung tissues, and might cause genomic instability and mutation [35]. The ATR-P53-mediated regulatory network maintains genome integrity, activating downstream genes whose products are involved in cell cycle progress and apoptosis. Although P53 and P21^WAF1^ upregulation arrested the cell cycle at G1 or G2/M checkpoints, we observed delayed progression in S, but not G1 phase. Previous studies demonstrated similar results [16, 17, 30, 33], implying that S phase arrest might be a general response to carcinogenic active PAH metabolites. P53-induced cell apoptosis is another response to DNA damage. Bax is a P53 trans-activation target involved in mitochondria-associated apoptosis activation. With acute PAH exposure, P53 and Bax were upregulated in a dose-dependent manner, and cells with irreparable DNA damage underwent apoptosis. Although the DDR coordinates DNA repair with cell cycle progression and apoptosis induction, aberrant cells might occasionally survive due to DDR network perturbation, leading to genomic mutations. With chronic PAH exposure, activation of PAH metabolic enzymes enabled cells to transform the PAHs into PAH-ROS more efficiently. These ROS induced genomic instability via their reactive electrophilic groups, causing DNA damage and increasing micronucleus frequencies. To counteract mutation risk, 10 G cells activated DDR regulators and their downstream genes, leading to prolonged cell cycle arrest and increased apoptosis. However, the key regulator of DDR, P53 expression was irregular, possibly perturbing the DDR. By further acute PAH exposure, AKR expression in 10 G cells was increased. The activated PAH metabolic phenotype and aberrant metabolic pathways perturbed the cellular redox status, further increasing oxidative damage in these cells. Although ATR expression was positively with the extent of DNA damage, P53 level was not coordinated with the transduced DNA damage signal. P53 was downregulated, especially at high PAH levels, while P21^WAF1^ was upregulated, leading to significant S phase arrest. S phase arrest is a possible mechanism to cope with DNA lesions that have escaped the G1 checkpoint [35, 36]. However, inappropriate S phase entry can lead to DNA damage accumulation during replication and mitosis. It appeared that significant DNA damage and cell cycle arrest promoted apoptosis in PAH-exposed cells, however the P53-Bax pro-apoptotic mechanism was inhibited due to P53 and Bax dysregulation. Although 10 G cells had higher mortality rates, the number of apoptotic cells decreased and more dead cells exhibited necrotic characteristics. Apoptosis inhibition resulting from DDR perturbation could permit the survival or continued growth of cells with genomic abnormalities, thereby enhancing the chance of mutagenic transformation.

In summary, we characterized long-term atmospheric PAH concentrations during haze and non-haze episodes. We assessed PAH-induced physiopathological alterations in two human lung cell lines for the first time. We found that chronic low-dose PAH exposure saturated xenobiotic metabolic pathways, increasing ROS production. Chronic ROS stress induced DNA damage and genomic instability, resulting in DDR network perturbation. Dysregulation of PAH metabolism and the DDR altered cellular homeostasis and increased cell susceptibility to subsequent PAH exposures, enhancing the likelihoods of mutation and pathological transformation.

## MATERIALS AND METHODS

### Chemicals and reagents

The EPA16 PAHs standard reagent, dimethyl sulfoxide (DMSO), ethylene diamine tetraacetic acid (EDTA), and thiazolyl blue tetrazolium bromide (MTT) were purchased from Sigma (St. Louis, USA). The n-hexane and dichloromethane were of high-performance liquid chromatography (HPLC) grade. Triton X-100, and Tris-HCl were purchased from Aladdin Reagent Co., Ltd (Shanghai, China). The propidium iodide (PI) cell-cycle kit, Annexin V-FITC apoptosis detection kit, protein extraction kit, SDS-PAGE, PVDF membrane, and antibodies (primary and secondary) were provided by KeyGen Biotech (Nanjing, China).

### PM2.5 sampling and sample treatment

A PM2.5 monitoring and sampling program was carried out at Xixi Campus, Zhejiang University in Hangzhou, China, during August (non-haze episode) and November 2015 (haze episode). The real-time PM2.5 concentration was measured and recorded using a Dust Trak II 8530 Aerosol Monitor (TSI, USA). PM2.5 was sampled using a BR 2010-S PM2.5 sampling system (Borain Electronic Instrument Company, Qingdao, China). The volume of filtered air was approximately 360 m^3^/filter with a sampling rate of 1.05 m^3^/min. Ambient PM2.5 was continuously collected onto glass fiber filters (8×10 inches, MUNKTELL Inc., Sweden). After sampling, filters were wrapped with cleaned aluminum foil and stored at -20°C.

Sample extractions were performed according to the United States Environmental Protection Agency (US EPA) Method TO-13A norm (US EPA, 1999). Each sample was extracted with 125 mL dichloromethane for at least 20 h at 4 cycles/h in a Soxhlet extractor, then evaporated to dryness, reconstituted with n-hexane, and concentrated in a Kuderna-Danish concentrator tube. The extract was cleaned on a silica gel column and eluted with 15 mL n-hexane and 5 mL dichloromethane.

The eluent was then divided into two equal parts; the first was used for PAH analysis and the second for *in vitro* analyses. The PAH analysis sample was evaporated to dryness via a gentle stream of pure nitrogen gas, and then reconstituted in 1 mL n-hexane for subsequent analysis. The GC-MS configuration and temperature programs were set up according to Teixeira [37]. Quantification was performed using calibration curves (internal standard method). The sample for *in vitro* analyses was re-dissolved in DMSO and stored in the dark at -4°C.

### Cell culture and treatment

Human lung epithelial cell lines, A549 and NCI-H1975, were cultured in RPMI-1640 medium supplemented with 10% calf serum in humidified air containing 5% CO_2_ at 37°C. Cells were divided into two groups. Group 1 was cultured in RPMI-1640 for 48 h, and then directly exposed to PAH extracts at a maximum concentration of 4.0 μg/mL, corresponding to approximately 20 m^3^ of sampled air per mL. Group 2 cells were pre-treated in culture medium containing a low concentration of PAHs (0.02 μg/mL; benzo [a] pyrene content, 1–2 ng/mL) for 30 d. After pre-treatment, cells were washed and cultured without PAHs for 48 h, then further treated with PAH extracts (PAH concentration, 0.5–4.0 μg/mL; benzo [a] pyrene content, approximately 10 ng/mL). To quantify cellular uptake, 2 mL culture medium and 2 mL centrifuged sample supernatant were collected and diluted with acetonitrile to 10 mL. The solution was then centrifuged, filtered using a cellulose membrane (pore size 0.22 μm, Shanghai Xingya), and concentrated via gentle nitrogen flow to 2 mL. PAH content was measured using HPLC as previously described [38].

### Cell viability assay

Cell viability was measured using a Calcein-AM and PI double stain kit (Yisheng Biotech, China) according to the manufacturer’s instructions. Cells were seeded in 12-well culture plates and treated with PAHs. After 24-h treatment, cells were rinsed in PBS and incubated for 20 min in the dark at room temperature in 2 μmol/L Calcein-AM and 5 μmol/L PI. Cells were then washed three times in PBS. Under blue light, living cells appeared green and the nuclei of dead cells fluoresced red. Dead and living cells were sorted and counted via flow cytometry.

PAH cytotoxicities and IC50s were evaluated in cultured cells via MTT assay according to Mosmann [39]. In brief, cells were seeded at 1×10^4^ cells/well in 96-well plates for 18 h at 37°C with 5% CO_2_, and then incubated in medium containing PAHs for 24 h. Medium was then replaced with 0.1 mL serum-free medium containing MTT (0.5 mg/ml final concentration) and incubated for 4 h. Finally, medium was replaced with 0.1 mL DMSO and measured spectrophotometrically in an ELISA plate reader (Model 550, Bio-Rad) at a wavelength of 570 nm. Relative cell growth (%) compared to control cells cultured in media without PAHs was calculated as follows:

V%=([A]experimental–[A]blank)/ ([A]control–[A]blank)×100%

### Intracellular ROS measurement

Intracellular ROS accumulation was detected using a reactive oxygen species assay kit (Beyotime, China) according to the manufacturer’s instructions. A total of 1×10^6^ cells were collected and washed twice with PBS. Cells were stimulated with medium containing 10 μM DCFH-DA for 20 min at 37°C. After removing the medium and washing cells with serum-free medium, intracellular ROS levels were observed under a fluorescence microscope. Cells were then collected, and fluorescence intensity was examined using flow cytometry.

### Comet assay

The Comet assay was performed as described by Singh, *et al.* [40]. Briefly, cells were seeded in 6-well plates at 2×10^5^ cells/well and then treated with PAHs for 24 h. Treated cells were collected, mixed with 100 μL 0.5% low melting point agarose (Sigma), and quickly poured onto a microscope slide coated with 50 μL 1% normal melting point agarose (Sigma). After solidification, slides were immersed in ice-cold lysis solution (2.5 M NaCl, 100 mM disodium EDTA, 1.2% Tris) for 2 h in the dark. 1% Triton X-100 and 10% DMSO were added to lysed cells to allow DNA unfolding. Slides were then fixed within a horizontal gel electrophoresis tank and immersed in electrophoresis buffer. After 1 h of DNA denaturation, electrophoresis was carried out for 20 min at 1V/cm (300mA). Slides were then rinsed gently three times with neutralization buffer. Comets captured from each slide were examined using fluorescence microscopy.

### Micronucleus assay

Micronuclei can be observed in binucleated cells that complete nuclear division and are blocked from cytokinesis using cytochalasin-B. Treated cells were washed, incubated with 4.5 μg/mL cytochalasin-B (Sigma) for 24 h, and harvested. Cells were then washed with PBS, treated with 0.05% KCl for 3 min at room temperature, and fixed in methanol/acetic acid (5:1, v/v) for 15 min. Cells were stained with DAPI in PBS and mounted for immunofluorescence observation. The frequency of micronuclei was assessed in 2,000 binucleated cells/treatment using an inverted microscope.

### Cell cycle and apoptosis assays

For the cell cycle assay, 5×10^5^ cells/well were seeded in 6-well plates, collected, washed three times with phosphate buffered saline (PBS), and fixed with 75% ice-cold ethanol for 24 h at 4°C. Fixed cells were washed with PBS and then stained with 100 μl RNase A and 100 μl PI sequentially. After incubation for 30 min at 37°C, samples were analyzed via flow cytometry (Caliber, CA, USA). Apoptosis was detected in cells prepared the same way using the Annexin V-FITC Apoptosis Detection Kit™ according to the manufacturer’s protocol (BD Pharmingen, San Diego, CA). Each experiment was repeated at least three times.

### Quantitative real time PCR and western blotting

Total RNA was extracted using a total RNA miniprep kit (Sigma) and digested with DNase I. cDNA was synthesized using oligo-dT and random hexamer primers for SYBR Green qPCR supermix-UDG kit (Invitrogen, Carlsbad, USA) analysis according to the manufacturer’s protocols. Triplicate biological samples were obtained for PCR analysis. PCR primers were designed and checked using LAST (NCBI), and PCR products were limited to 100–200 bp in length.

Proteins were extracted from treated cells and total protein was quantified using the BCA protein assay kit (Promega, USA). Equal protein amounts were separated via SDS-PAGE, transferred onto nitro-cellulose membranes, blocked, and incubated overnight with monoclonal antibodies. After washing, membranes were incubated with an HRP-conjugated secondary antibody for 2 h at room temperature. Bands were visualized using the Westzol enhanced chemiluminescence kit (Intron, Sungnam, Korea) and expression was normalized to the housekeeping gene.

### Statistical analysis

All data are expressed as means ± standard deviation (SD) of three independent repetitions as analyzed using Student’s t-test. P<0.05 was considered significantly different.

## References

[1] Andreae MO, Jones CD, Cox PM (2005). Strong present-day aerosol cooling implies a hot future. Nature.

[2] Luke C, Rea W, Smith-Willis P, Fenyves E, Pan Y (2006). Adverse health effects of outdoor air pollutants. Environment International.

[3] Kan HD, Chen RJ, Tong SL (2012). Ambient air pollution, climate change, and population health in China. Environment International.

[4] McMurry PH (2000). A review of atmospheric aerosol measurements. Atmospheric Environment.

[5] Zhou W, Tian DD, He J, Wang YM, Zhang LJ, Cui L, Jia L, Zhang L, Li LZ, Shu YL, Yu SZ, Zhao J, Yuan XY (2016). Repeated PM2.5 exposure inhibits BEAS-2B cell P53 expression through ROS-Akt-DNMT3B pathway-mediated promoter hypermethylation. Oncotarget.

[6] Poschl U (2005). Atmospheric aerosols: composition, transformation, climate and health effects. Angewandte Chemie International Edition English.

[7] Lewtas J (2007). Air pollution combustion emissions: characterization of causative agents and mechanisms associated with cancer, reproductive, and cardiovascular effects. Mutation Research.

[8] Poirier MC (2004). Chemical-induced DNA damage and human cancer risk. Nature Reviews Cancer.

[9] Hecht SS (2003). Tobacco carcinogens, their biomarkers and tobacco-induced cancer. Nature Reviews Cancer.

[10] Moorthy B, Chu C, Carlin DJ (2015). Polycyclic aromatic hydrocarbons: from metabolism to lung cancer. Toxicological Sciences.

[11] Shimada T, Fujii-Kuriyama Y (2004). Metabolic activation of polycyclic aromatic hydrocarbons to carcinogens by cytochromes P450 1A1 and 1B1. Cancer Sciences.

[12] Nebert DW, Dalton TP (2006). The role of cytochrome P450 enzymes in endogenous signalling pathways and environmental carcinogenesis. Nature Reviews Cancer.

[13] Park JH, Mangal D, Tacka KA, Quinn AM, Harvey RG, Blair IA, Penning TM (2008). Evidence for the aldo-keto reductase pathway of polycyclic aromatic trans-dihydrodiol activation in human lung A549 cells. Proceedings of the National Academy of Sciences.

[14] Shiloh Y (2001). ATM and ATR: networking cellular responses to DNA damage. Current Opinion in Genetics & Development.

[15] Cimprich KA, Cortez D (2008). ATR: an essential regulator of genome integrity. Nature Reviews Molecular Cell Biology.

[16] Chung JY, Kim JY, Kim WR, Lee SG, Kim YJ, Park JE, Hong YP, Chun YJ, Park YC, Oh S, Yoo KS, Yoo YH, Kim JM (2007). Abundance of aryl hydrocarbon receptor potentiates benzo[a]pyrene-induced apoptosis in Hepa1c1c7 cells via CYP1A1 activation. Toxicology.

[17] Binkova B, Giguere Y, Rossner P, Dostal M, Sram RJ (2000). The effect of dibenzo[a,1]pyrene and benzo[a]pyrene on human diploid lung fibroblasts: the induction of DNA adducts, expression of p53 and p21(WAF1) proteins and cell cycle distribution. Mutation Research.

[18] Sancar A, Lindsey-Boltz LA, Unsal-Kacmaz K, Linn S (2004). Molecular mechanisms of mammalian DNA repair and the DNA damage checkpoints. Annual Review of Biochemistry.

[19] Riley T, Sontag E, Chen P, Levine A (2008). Transcriptional control of human p53-regulated genes. Nature Reviews Molecular Cell Biology.

[20] Ko CB, Kim SJ, Park C, Kim BR, Shin CH, Choi S, Chung SY, Noh JH, Jeun JH, Kim NS, Park R (2004). Benzo(a)pyrene-induced apoptotic death of mouse hepatoma Hepa1c1c7 cells via activation of intrinsic caspase cascade and mitochondrial dysfunction. Toxicology.

[21] de Kok TM, Driece HA, Hogervorst JG, Briede JJ (2006). Toxicological assessment of ambient and traffic-related particulate matter: a review of recent studies. Mutation Research.

[22] Lin CC, Chen SJ, Huang KL, Lee WJ, Lin WY, Tsai JH, Chaung HC (2008). PAHs, PAH-induced carcinogenic potency, and particle-extract-induced cytotoxicity of traffic-related nano/ultrafine particles. Environmental Science & Technology.

[23] Tapanainen M, Jalava PI, Maki-Paakkanen J, Hakulinen P, Happo MS, Lamberg H, Ruusunen J, Tissari J, Nuutinen K, Yli-Pirila P, Hillamo R, Salonen RO, Jokiniemi J (2011). *in vitro* immunotoxic and genotoxic activities of particles emitted from two different small-scale wood combustion appliances. Atmospheric Environment.

[24] Tsai JH, Chen SJ, Huang KL, Lin TC, Chaung HC, Chiu CH, Chiu JY, Lin CC, Tsai PY (2012). PM, carbon, PAH, and particle-extract-induced cytotoxicity emissions from a diesel generator fueled with waste-edible-oil-biodiesel. Aerosol and Air Quality Research.

[25] Yunker MB, Macdonald RW, Vingarzan R, Mitchell RH, Goyette D, Sylvestre S (2002). PAHs in the Fraser River Basin: a critical appraisal of PAH ratios as indicators of PAH source and composition. Organic Geochemistry.

[26] Mishra N, Ayoko GA, Morawska L (2016). Atmospheric polycyclic aromatic hydrocarbons in the urban environment: occurrence, toxicity and source apportionment. Environmental Pollution.

[27] Carreras HA, Calderon-Segura ME, Gomez-Arroyo S, Murillo-Tovar MA, Amador-Munoz O (2013). Composition and mutagenicity of PAHs associated with urban airborne particles in Cordoba, Argentina. Environmental Pollution.

[28] Cimino F, Speciale A, Siracusa L, Naccari C, Saija A, Mancari F, Raciti R, Cristani M, Trombetta D (2014). Cytotoxic effects induced *in vitro* by organic extracts from urban air particulate matter in human leukocytes. Drug Chemical Toxicology.

[29] Vanden Berghe T, Linkermann A, Jouan-Lanhouet S, Walczak H, Vandenabeele P (2014). Regulated necrosis: the expanding network of non-apoptotic cell death pathways. Nature Reviews Molecular Cell Biology.

[30] Palackal NT, Lee SH, Harvey RG, Blair IA, Penning TM (2002). Activation of polycyclic aromatic hydrocarbon trans-dihydrodiol proximate carcinogens by human aldo-keto reductase (AKR1C) enzymes and their functional overexpression in human lung carcinoma (A549) cells. Journal of Biological Chemistry.

[31] Santaguida S, Amon A (2015). Short- and long-term effects of chromosome mis-segregation and aneuploidy. Nature Reviews Molecular Cell Biology.

[32] Fei PW, El-Deiry WS (2003). P53 and radiation responses. Oncogene.

[33] Black KA, McFarland RD, Grisham JW, Smith GJ (1989). S-phase block and cell death in human lymphoblasts exposed to benzo[a]pyrene diol epoxide or N-acetoxy-2-acetylaminofluorene. Toxicology and Applied Pharmacology.

[34] Shimada T (2006). Xenobiotic-metabolizing enzymes involved in activation and detoxification of carcinogenic polycyclic aromatic hydrocarbons. Drug Metabolism and Pharmacokinetics.

[35] Wang XW, Harris CC (1997). p53 tumor-suppressor gene: clues to molecular carcinogenesis. Journal of Cellular Physiology.

[36] Branzei D, Foiani M (2008). Regulation of DNA repair throughout the cell cycle. Nature Reviews Molecular Cell Biology.

[37] Teixeira EC, Agudelo-Castaneda DM, Fachel JM, Leal KA, Garcia KD, Wiegand F (2012). Source identification and seasonal variation of polycyclic aromatic hydrocarbons associated with atmospheric fine and coarse particles in the Metropolitan Area of Porto Alegre, RS, Brazil. Atmospheric Research.

[38] Bai HZ, Zhou J, Zhang HJ, Tang GP (2017). Enhanced adsorbability and photocatalytic activity of TiO2-graphene composite for polycyclic aromatic hydrocarbons removal in aqueous phase. Colloids and surfaces B-Biointerfaces.

[39] Mosmann T (1983). Rapid colorimetric assay for cellular growth and survival-application to proliferation and cyto-toxicity assays. Journal of Immunological Methods.

[40] Singh NP, McCoy MT, Tice RR, Schneider EL (1988). A simple technique for quantitation of low-levels of DNA damage in individual cells. Experimental Cell Research.

